# Enhancement of Conversion Efficiency of B(In)GaAs Solar Cells: A Simulation and Numerical Investigation

**DOI:** 10.3390/ma19204271

**Published:** 2026-10-09

**Authors:** Dalanda Slimi, Faouzi Saidi, Abdelhamid Helali, Abdelkader Aissat, Mohamed Houcine Dhaou, Mohamed Alaaedine Madani, Demir Ilkay

**Affiliations:** 1 Laboratory of Micro-Optoelectronics and Nanostructures (LR99ES29), Faculty of Sciences Monastir, University of Monastir, Environment Avenue, Monastir 5019, Tunisia; slimidalanda123@gmail.com (D.S.); abdelhamid.helali@gmail.com (A.H.); 2 Institut Supérieur des Sciences Appliqués et Technologie de Sousse, Université de Sousse, Sousse 4011, Tunisia; 3 Department of Material Science, Faculty of Material Science, Mathematics and Computer Science, University of Ahmed Draia Adrar, Adrar 01000, Algeria; 4 Department of Physics, College of Science, Qassim University, P.O. Box 6644, Buraydah 51452, Almolaydah, Saudi Arabia; 5 Laboratory for the Development of Renewable Energies and Their Applications in Saharan Areas (LDREAS), Tahri Mohamed University of Bechar, Bechar 08000, Algeria; madani.mohammed@univ-bechar.dz; 6 Sivas Cumhuriyet University Nanophotonics Application and Research Center-CÜNAM, Department of Nanotechnology Engineering, Sivas Cumhuriyet University, 58140 Sivas, Turkey

**Keywords:** materials, semiconductors, thin film, solar cell, optical proprieties

## Abstract

This study focuses on the modeling and optimization of B_x_Ga_1−x_As/In_y_Ga_1−y_As double-junction photovoltaic cells and GaAs/B_x_In_y_Ga_1−x−y_As quantum well pin cells. It relies on Silvaco Atlas software 2.10.26R to simulate these structures, considering the desired number of QWs or the constant percentage of boron and indium in the III-V-based alloys of B_x_Ga_1−x_As, In_y_Ga_1−y_As, and B_x_In_y_Ga_1−x−y_As. The B_x_In_y_Ga_1−x−y_As material finds its application in the fabrication of quantum wells incorporated into the intrinsic GaAs-type absorber layer. This absorber layer is positioned between the heavily doped base and the emitting regions, in order to generate a uniform field that allows all photogenerated carriers to escape from the QWs. As part of the modeling, a comparison was made between a 40-layer GaAs/B_x_In_y_Ga_1−x−y_As quantum well solar cell and a standard GaAs solar cell. This analysis revealed an increase in the conversion efficiency of the simulation results from 22.66% to 31.73%. As a result, the integration of quantum wells into the structure of solar cells seems promising to optimize their performance. The optimized B_x_Ga_1−x_As/In_y_Ga_1−y_As tandem structure demonstrated a short-circuit current density of 17.50 mA/cm^2^, an open-circuit voltage of 1.99 V, a fill factor of 87.29%, and a conversion efficiency of 30.40% under AM1.5G illumination at 300 K.

## 1. Introduction

In the global energy landscape, fossil fuels (coal, oil, and gas) hold a considerable market share, making them a predominant sector in the worldwide generation of energy. The reserves of these non-renewable fossil fuel sources, which have adverse environmental effects, are still viable for approximately a few decades [1]. As soon as the availability of conventional energy resources is compromised [2], human society will be forced to seek alternative solutions to ensure its energy production. This approach falls within the scope of renewable energy, which encompasses hydroelectric, geothermal, wind, biomass, and photovoltaic energy sources. The energy sources in question are derived either directly or indirectly from solar energy.

According to studies, 0.1% of the solar energy incident on the Earth’s surface is enough to meet the current global energy demand. The technology employed to generate this energy is referred to as a “photovoltaic cell” [3]. These devices are semiconductor-based systems that facilitate the conversion of light into useful electrical energy. The remarkable growth in solar technology research, marked by the creation of the first high-efficiency solar cell, has led to a significant increase in the efficiency of solar cells [4].

The development of semiconductor thin-film technology has enabled the replacement of bulk materials with thin films in photovoltaic devices, leading to significant reductions in manufacturing cost and device weight. In addition, thin-film technologies allow growth on flexible substrates, eliminating the need for expensive bulk crystalline wafers such as c-Si [5,6] thin film.

Currently, research in this field, which is characterized by abundance and great diversity, focuses mainly on the advantages of multi-junction cells and quantum well cells.

Multi-junction photovoltaic architectures provide a viable route to surpass the Shockley–Queisser efficiency limit inherent to single-junction solar cells [4]. By stacking sub-cells with progressively narrower band gaps, these tandem configurations exploit the solar spectrum more effectively, achieving theoretical efficiencies exceeding 50% under concentrated illumination. However, this performance hinges on precise band gap tuning and rigorous current matching across sub-cells.

It has been shown that the efficiency of these solar cells improves over time, thanks to the increase in the number of junctions or wells. In addition, a new avenue for improving cell efficiency is the use of alloy-based materials with different gaps, such as gallium arsenide (GaAs). Furthermore, GaAs thin film, a crystalline compound of (Ga) and (As), is a particularly relevant subject of study in materials physics. This material is distinguished by its ability to offer significant resistance to heat and radiation, which are two factors that can compromise the efficiency of solar cells. Although the cost of a GaAs solar module is high, it is important to emphasize that it holds the world record for efficiency, with a rate exceeding 30%. In the context of the space industry, the emphasis on energy efficiency rather than minimizing the costs of electricity production in space gives particular importance to multi-junction GaAs cells. These are preferred for solar power generation applications such as concentrated solar power (CSP) structures and solar power systems onboard spacecraft. Their ability to operate at extremely high temperatures, far exceeding those reached by c-Si-based solar systems [7,8], makes them particularly well-suited to these harsh environments.

GaAs solar cells have played a pivotal role as concentrators and space-based solar cells [9], paving the way for new market opportunities. These technological advances could foster the growth of large-scale power systems and electric vehicles powered by photovoltaic cells, which are two rapidly expanding sectors. GaAs single-junction solar cells, which are essential sub-cells for multi-junction photovoltaic cells, have achieved an exceptional efficiency of 29.1%, which is the highest ever recorded for a single-junction photovoltaic cell excited by the AM1.5 standard spectrum. The development of multi-junction GaAs solar cells (comprising three or more junctions) has been driven by the need to improve the efficiency of single-junction photovoltaic cells, which have proven to be limited by the Shockley–Queisser limit. GaAs-based solar cells with multiple junctions are expected to find extensive application in the space sector due to their high photovoltaic conversion capacity.

Recent advances in III-V tandem solar cells have demonstrated significant progress, with record efficiencies reaching 33.39% for GaInP/quantum well tandem cells [10], over 32.23% for GaAsP/Si tandem cells [11] and Si quantum well cells, and over 32.23% for GaAsP/Si tandem cells.

The integration of quantum well structures has proven particularly promising for improving photovoltaic performance, as demonstrated by GaAs/InAs quantum wells [12]. These advances highlight the continued importance of computational modeling and optimization studies in advancing III-V solar cell technology.

Quantum well solar cell (QWSC) applications have generated significant interest. They are characterized by the integration of alternating layers of quantum wells (QWs) and quantum barriers (QBs) within the involved region of a PIN-based solar cell [8]. This innovative structure, composed of several wide-band gap (barrier) and narrow-band gap (well) semiconductors, aims to optimize the spectral response below the absorption threshold of the barrier material. The crucial challenge lies in optimizing the number, thickness, and composition of the structural parameters to improve photocurrent generation while eliminating recombination losses. Multi-junction solar cells (MJSCs), equipped with quantum well super-arrays, exhibit optimized efficiencies of 39.5% and 34.2% under excitation of AM1.5 and AM0 standard spectrum, respectively [13].

However, the experimental optimization of solar cell structures poses a significant challenge due to their intrinsic complexity, high cost, and lengthy development time. In the context of solar cell design, numerical modeling and simulation are useful tools for optimizing their structure. These methods enable an understanding of the main physical phenomena and behaviors, resulting in reduced development time and cost. In research into tandem solar cells with a B_x_Ga_1−x_As/In_y_Ga_1−y_As double-junction and p-i-n solar cells with quantum wells (GaAs/B_x_In_y_Ga_1−y_As), it is necessary to optimize the anti-reflective layer, tunnel junction, and well width for different barrier thicknesses. This approach allows for higher conversion efficiency. Consequently, detailed numerical modeling of tandem solar cells and quantum well cells is imperative.

Within the context of the ongoing evolution of our conventional photovoltaic technologies, we are currently focused on optimizing performance and exploring new materials to maximize energy conversion. Relevant studies, notably those by Suchikova et al. (2025) [14] and Al-Ezzi and Ansari (2022) [15], have made significant contributions in this area by proposing innovative approaches to overcome the current limitations of solar cells. Suchikova et al. (2025) [14] focused on exploring nanostructures and their applications in improving photovoltaic efficiency, while Al-Ezzi and Ansari (2022) [15] conducted in-depth analyses of conversion mechanisms and optimization strategies for solar devices. This work highlights the importance of continuous innovation in this sector. It also allows us to position the present study within the context of the latest scientific advances, identifying both gaps to be filled and opportunities for improving existing photovoltaic technologies.

## 2. Device Structure and Material

Silvaco ATLAS was selected as the simulation platform for this study because it meets several essential criteria for the accurate modeling of III-V quantum well and multi-junction photovoltaic devices, including:

ATLAS uses comprehensive drift-diffusion and quantum mechanics models specifically calibrated for III-V semiconductors. In the Silvaco library, users have access to validated material parameters for GaAs and InGaAs. These parameters ensure accurate prediction of electrical and optical properties, thus optimizing the performance of the finished products. Through a process of continuous improvement, the simulator has been optimized to incorporate advanced features for handling carrier confinement, quantum tunneling, and escape mechanisms in QW structures. The software integrates absorption, reflection, and generation profiles that vary with wavelength. These features are essential for analyzing solar cells using standard solar spectra (AM1.5G).

As part of this project, the use of four distinct semiconductors was studied: GaAs, InGaAs, BGaAs, and finally BInGaAs. GaAs-based photovoltaic cells are therefore considered a promising technology for high power conversion efficiency. In the context of high-concentration photovoltaic technologies, the choice of the base material is of crucial importance. In the classification of semiconductors, gallium arsenide, denoted GaAs, occupies a specific position as a direct band gap material. This property distinguishes it by its remarkably high absorption capacity in the full range of the solar spectrum, thus giving it a significant role in photovoltaic and optoelectronic applications.

In light of these advances, Alta Devices recently achieved an efficiency of 28.8% with a GaAs single-junction solar cell, thus setting a benchmark in terms of photovoltaic efficiency [16]. When selecting a material, InGaAs should be considered a semiconductor with an energy band gap that is susceptible to fluctuations of 1.42 eV relative to GaAs and 0.36 eV relative to InAs, with fluctuations in the In/Ga ratio.

Exploiting the near-perfect lattice match and the absence of recombination at the interface states of the InGaAs/GaAs isotype junction has enabled the elimination of front-surface recombination losses at the heterojunction interface and, consequently, the optimization of solar cell efficiency [17]. As the spectral range of currently produced solar cells expands and their efficiency increases, the use of materials with a 1.25 eV or 1 eV band gap, which are lattice-matched with GaAs, is essential for the production of next-generation solar cells.

Extensive research has been conducted on the engineering of GaInAsN-based photovoltaic cells, or more recently, BInGaAs-based photovoltaic cells, to explore their potential for solar power generation. Solar cells have been developed from both materials, and the quantum efficiency of BinGaAs-based solar cells has been found to be higher than that of InGaAsN-based cells. These higher efficiencies are the result of increased carrier mobility within the BinGaAs alloy compared to InGaAsN. This observation could be attributed to a low defect density in the BinGaAs material [18].

The GaAs and InGaAs material files are included in the TCAD libraries. Thus, it appears that the B_x_Ga_1−x_As and B_x_In_y_Ga_1−x−y_As materials, which have been little studied so far, are not yet included in commercial TCAD programs. Therefore, the researchers developed the files for these materials based on the best estimates from the literature for parameters such as band gap, mobility, polarization coefficient, and optical absorption coefficients. This work follows previous research on the subject. A few years ago, interest in BgaAs and BinGaAs alloys, which are potential candidates for use in multi-junction solar cells and detectors, was rekindled.

**Figure 1:**
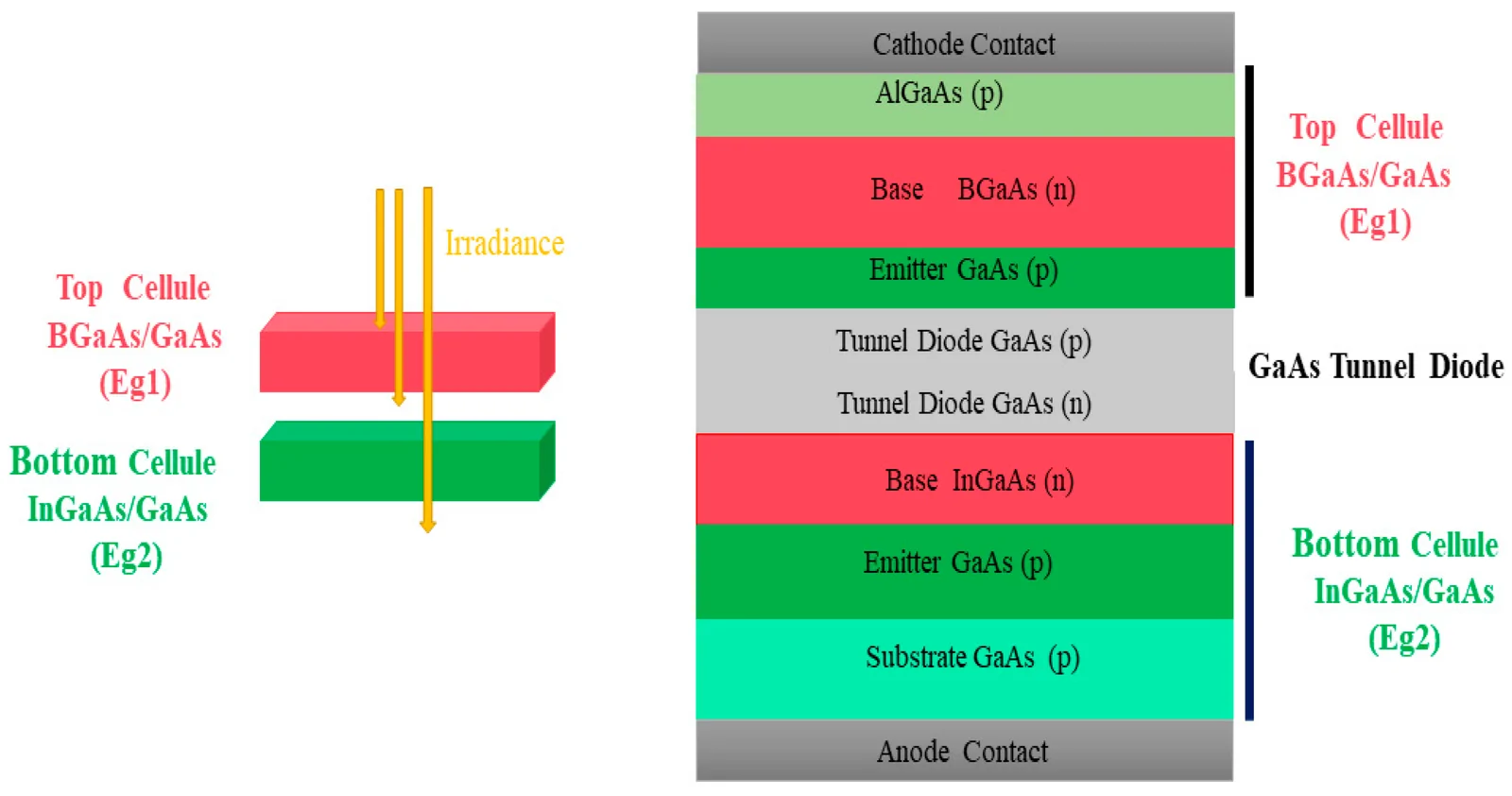
Schematic structure of DJ B_x_Ga_1−x_As/In_y_Ga_1−y_As tandem solar cells.

The solar cell under investigation in this research was a double-junction (MJsc) device consisting of an upper BgaAs/GaAs cell with a band gap (Eg) ranging from 1.1 to 1.42 eV, which was electrically and optically interconnected in series with a lower InGaAs/GaAs cell through an ab initio tunnel junction (see Figure 1). Each component is meticulously designed with the aim of optimizing the device’s performance. A thorough examination of the configuration of BgaAs/InGaAs double-junction solar cells reveals that their design is meticulously crafted to optimize two distinct yet interconnected aspects: light absorption and charge carrier transport.

**Figure 2:**
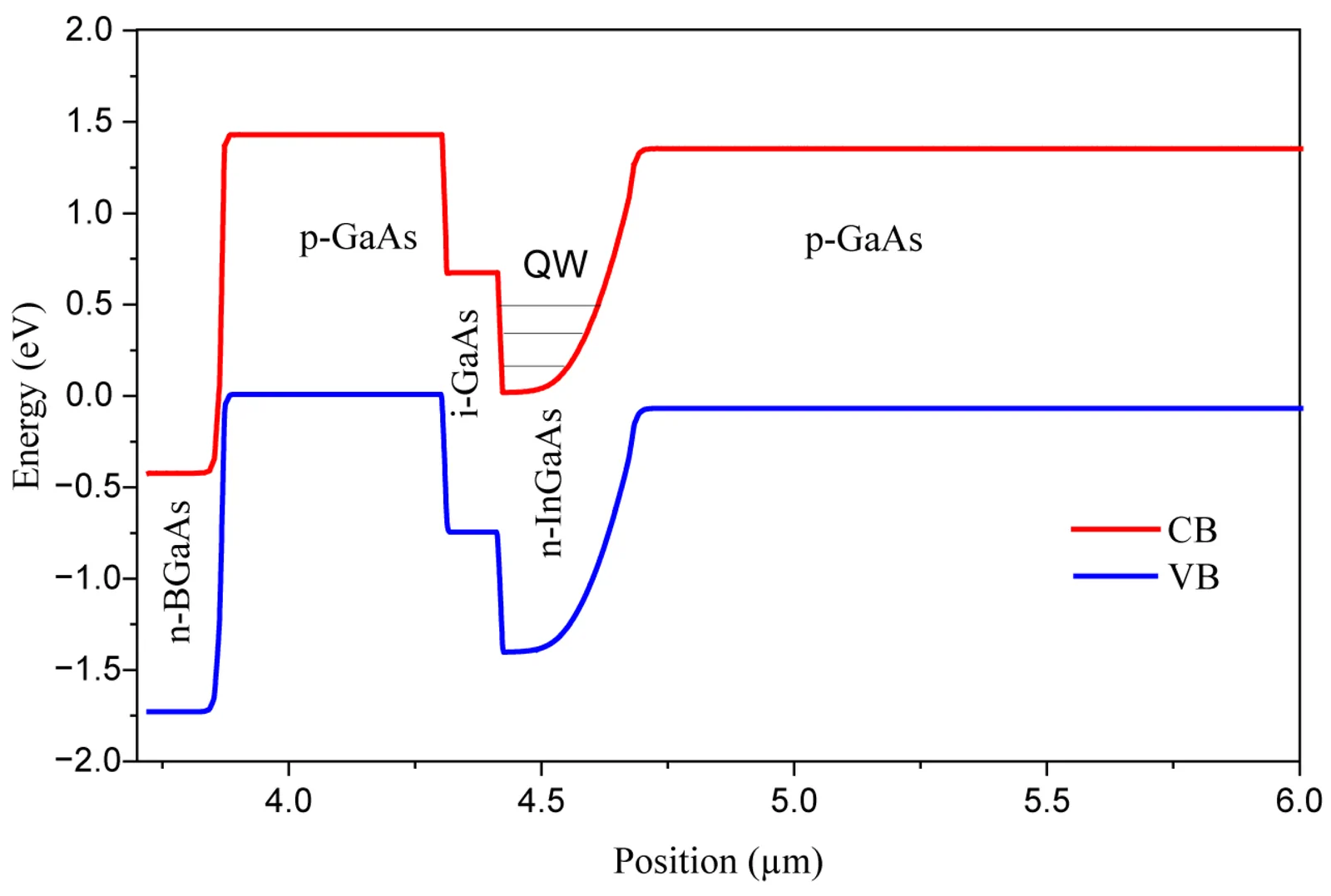
Diagram of the gradual band structure of the BgaAs/InGaAs tandem cell (DJSC).

As shown in the energy band diagram of the DJSC BGaAs/InGaAs cell under zero bias voltage conditions (see Figure 2), the system’s efficiency is optimal. In our analysis, it was determined that E_g2_ and E_g1_ represent the band gaps of the lower and upper sub-cells, respectively. These absorption thresholds are key indicators for our evaluation.

The upper sub-cell is transparent to incident photons with energy values below the band gap (E_g1_). These photons continue their trajectory toward the lower sub-cell or leave the DJSC. In our study, we found that the photons absorbed in the lower sub-cell are those whose energy is between the band gap energy of the upper sub-cell and that of the lower sub-cell (E_g2_ < E_ph_ = hν < E_g1_). For our analysis, it is essential to understand that this substance is transparent to all photons with energy equal to E_ph_ = hν < E_g2_.

The energy diagram of the DJSC cell illustrates the space charge region, the quasi-neutral region, and the energy levels of the conduction (E_c_) and valence (E_v_) bands. It should be noted that the actual band curvature may vary slightly due to dopant diffusion, particularly in tunnel diode regions. As the study results reveal, the integration of Al_x_Ga_1−x_As window layers, with a band gap energy of 1.54 eV, optimizes transparency and simplifies emitter passivation. Consequently, these effects are likely to affect minority carrier performance, resulting in a decrease in surface recombination effects.

**Figure 3:**
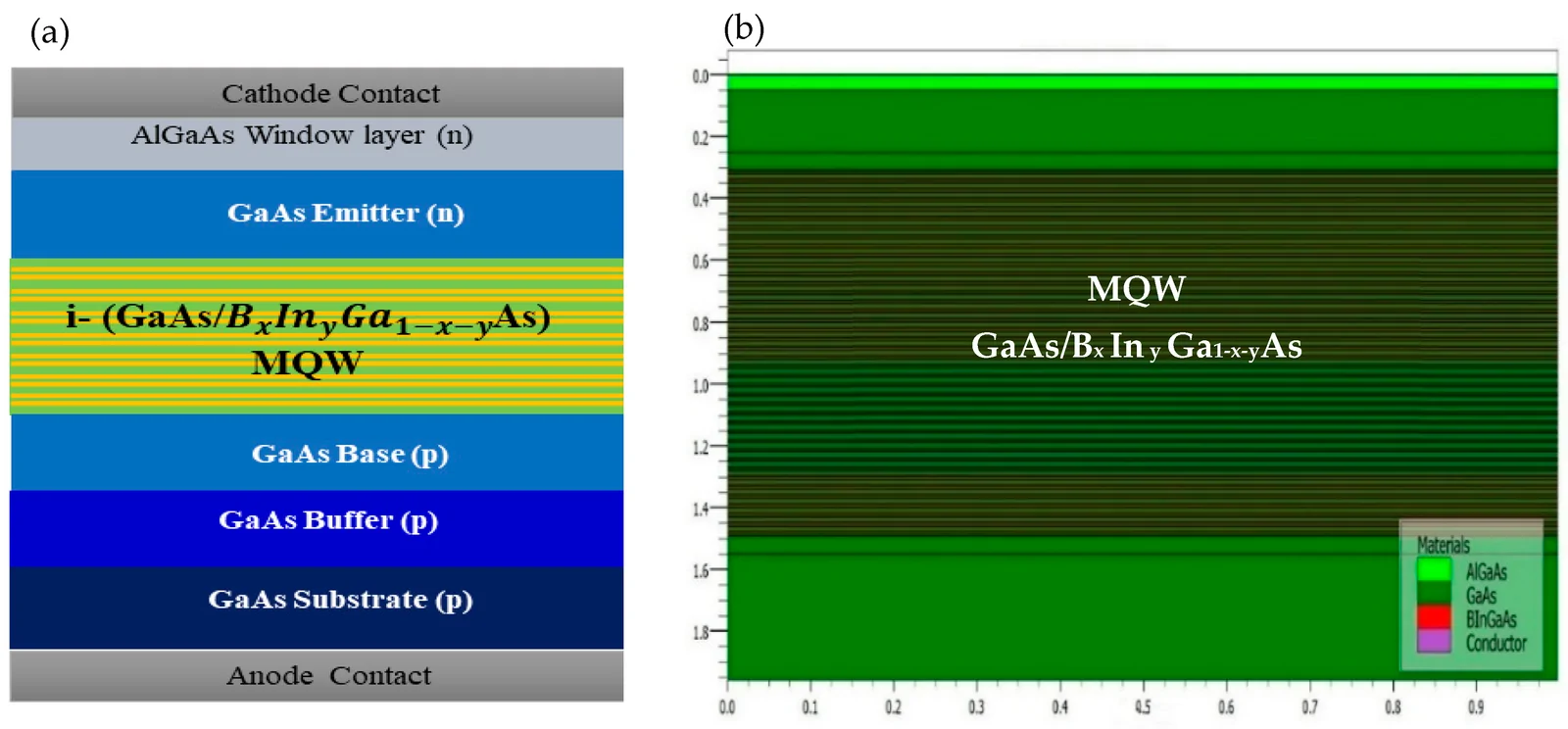
(**a**) Structure of multiple quantum well (MQW) pin solar cells and (**b**) zoomed view of the proposed QWSC.

The second type of solar cell studied is a generalized GaAs/B_x_In_y_Ga_1−x−y_As MQWSC structure based on a p-i-n junction, as shown in Figure 3a, which consists of six different layers: the Al_x_Ga_1−x_As window, the GaAs emitter, the absorber (in which the GaAs/B_x_In_y_Ga_1−x−y_As quantum wells are inserted), the GaAs base, the BSF (Back Surface Field) layer, and the substrate.

The constituent materials of these layers have distinct bandwidths. Quantum wells are inserted within the intrinsic layer, which is located between the p- and n-type regions. The active optical absorption region consists of 40 periods of GaAs/B_x_In_y_Ga_1−x−y_As MQWs. The thickness of the barrier was meticulously designed to be relatively thin in order to optimize current routing between the quantum wells and to increase the collection probability of photogenerated carriers.

The structure of the GaAs/B_x_In_y_Ga_1−x−y_As quantum wells solar cell was studied to demonstrate the impact of their insertion on the characteristic parameters of the solar cell. The introduction of quantum wells leads to the creation of numerous confined electron and hole states within the intrinsic region, thereby enhancing carrier drift by leveraging the inherent electric field of the p/n junction. Figure 3b shows the zoomed view of the QWSC to highlight the QWs in the intrinsic absorber layer.

**Figure 4:**
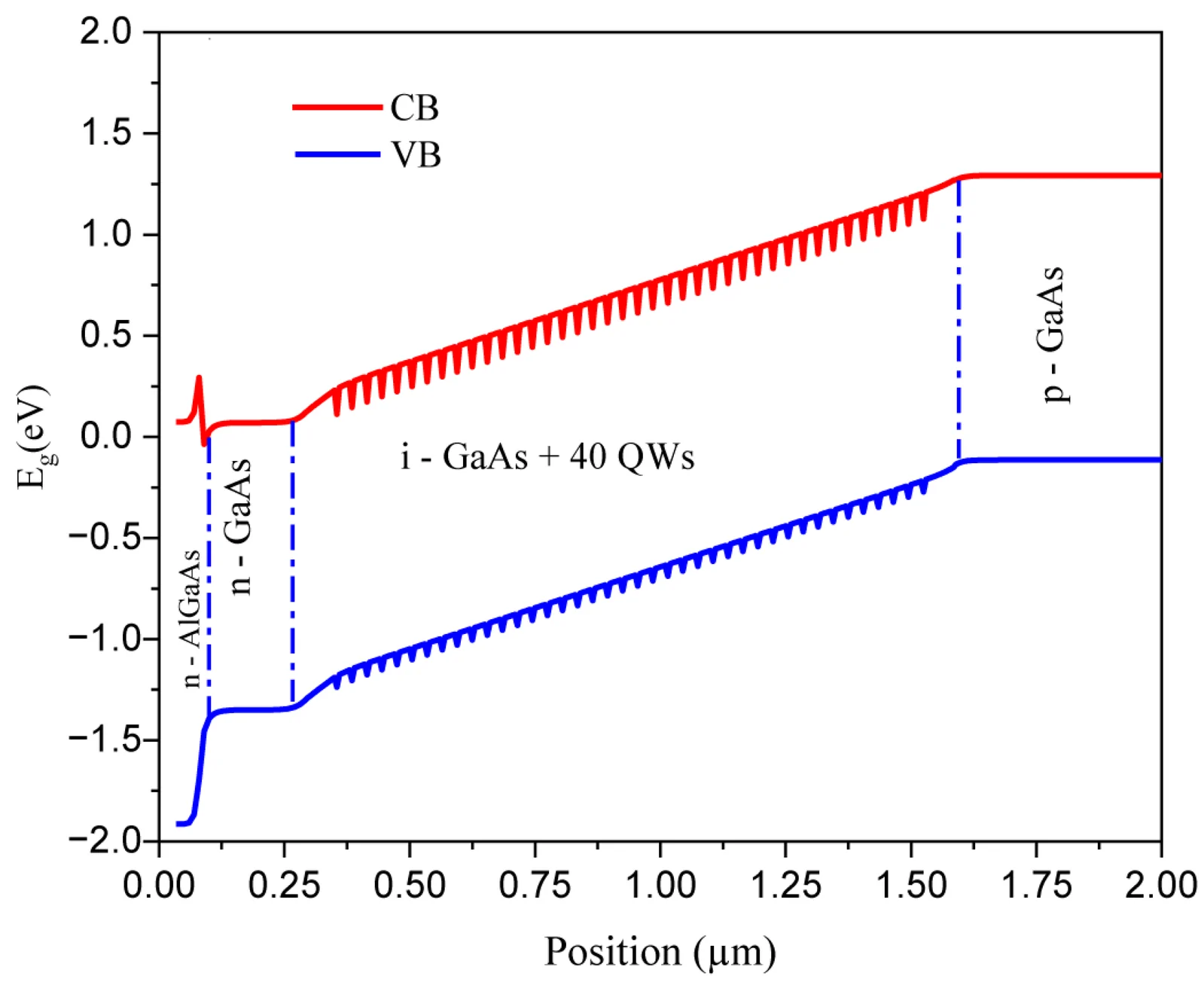
Energy band diagram in the quantum well region.

The energy band diagram in the MQW region, shown in Figure 4, demonstrates that the polarization-induced electric field inside the quantum well opposes the built-in electric field of the p-n junction. This increase in conductivity is accompanied by a decrease in the probability of carrier collection between quantum wells, resulting in a reduction in the photocurrent. Furthermore, quantum barriers trap photogenerated carriers in the wells.

In QWSCs, photoexcited carriers can escape from the QWs using thermal escape and tunneling mechanisms [19,20]. The experimental demonstration in [21] shows that the efficiency improvement of QWSCs compared to bulk cells, due to the photocurrent enhancement, is offset by the voltage loss in the QWs [22]. The thickness of the quantum well is carefully selected so that the band gap energy (E_a_) is narrower than that of the solar cell material (E_g_) [23]. In the case where the photon energy (hν) is higher than the excitation level energy (E_g_), the quantum well region behaves like a massive solar cell.

Indeed, for photons with energy lower than or equal to the quantum well energy (E_a_ < hν < E_g_), the latter can easily absorb the photons [24]. As a result, the overall photon current generated is high, leading to an increase in the short-circuit current. The material parameters employed in our simulation are enumerated in Table 1. In the case of a ternary alloy, the variation in the band gap energy with the composition can be described by an empirical quadratic expression (Thompson and Woolley formula) [25] of the type:

E_g_(x) = x·E_g1_ + (1 − x)·E_g2_ − b·x·(1 − x) (1)

In this relationship (1), x is the composition of the ternary; E_g1_ and E_g2_ are the band gap energies of the corresponding binaries. The parameter b, which quantifies the deviation from linearity, is called the band gap curvature parameter (bowing parameter).

In the study carried out on the InGaAs alloy, the evolution of its band gap energy could be represented by Equation (1), where the curvature parameter is set to 0.477 eV [26]. In the study of the B_x_Ga_1−x_As alloy, the evolution of the energy of its forbidden band can be described by a parabolic expression (1), with a curvature parameter of the order of 1.6 eV [27]:

E_g_ (B_x_Ga_1−x_As) = x·E_g_ (BAs) + (1 − x)·E_g_ (GaAs) − b·x·(1 − x) (2)

E_g_ (In_x_Ga_1−x_As) = x·E_g_ (InAs) + (1 − x)·E_g_ (GaAs) − b·x·(1 − x) (3)

where E_g_ (BAs) = 1.46 eV, E_g_ (InAs) = 0.36 eV, xbore = 0.08, and yinduim = 0.2.

**Table 1:** Major parameters used in the simulation [28,29,30,31,32,33].

Parameters	GaAs	B_x_Ga_1−x_As x = 0.08	Ga_y_In_1−y_As y = 0.2	QW B_x_In_y_Ga_1−x−y_As x = 0.08, y = 0.2
E_g_ (eV)	1.42	1.30	1.19	1.15
ε_r_	13.1	7.1119	14.20	13.00
τ_n_ (s)	1 ×10^−9^	1 × 10^−9^	1	1 × 10^−9^
τ_p_ (s)	2 × 10^−8^	1 × 10^−9^	1	1 × 10^−9^
χ (eV)	4.07	4.18	4.13	4.19
N_c_ (cm^−3^)	4.35 × 10^17^	1 × 10^17^	1.15 × 10^17^	2.13 × 10^18^
Nv (cm^−3^)	8.16 × 10^18^	5.99 × 10^18^	8.12 × 10^18^	8.48 × 10^18^
μ_n_ (cm^2^·V^−1^·s^−1^)	8800	7932	12,800	11,599
μ_p_ (cm^2^·V^−1^·s^−1^)	400	384	410	402.60

## 3. Results and Discussion

In the preceding section, a methodological framework was delineated. In this study, the results obtained during the simulation of BGaAs/InGaAs double-junction cells, as well as multiple quantum well PIN cells, are presented and analyzed in this section.

III-V multi-junction solar cells (MJSCs) are a product of excellence, composed of a multitude of cell junctions, or sub-cells, which form a high-performance assembly. The materials selected for the manufacture of the sub-cells are semiconductors from groups III and V, in accordance with the criteria established by our periodic table.

As part of our development strategy and our ongoing efforts to innovate and diversify, we have identified a wealth of opportunities with different secondary, ternary, and quaternary combinations of these materials. These will enable us to create sub-cells, opening up new avenues for the growth of our company. Our analysis has shown that the band gap and lattice constant can be adjusted by optimizing the composition of the material.

Research conducted as part of this study has led to the conclusion that the choice of semiconductor materials is crucial for optimizing yields. The energy gap between the two cells is a determining factor in this decision [34]. The optimal energy gap for single-junction solar cells, which gives them higher efficiency, is 1.35 eV for the upper BGaAs cell [35] and a wide range of 0.5 to 0.74 eV for the lower InGaAs cell [36]. The band gap is constant for both cells and is set at 1.30 eV and 1.19 eV for B_0.08_Ga_0.92_As/GaAs and GaAs/In_0.2_Ga_0.8_As cells, respectively. As part of our study, we analyzed the impact of semiconductor material selection on the performance of photovoltaic devices exposed to AM1.5G solar radiation.

DJ tandem solar cells, a recent innovation in the field of photovoltaics, use a tunnel junction between the two cells to avoid a parasitic junction, which is a fundamental concept in electronics and electricity. The MJSC concept has proven to be the optimal solution for achieving efficiencies above the S-Q limit [37] and, in addition, for reducing transmission and thermalization losses. This innovative approach consists of stacking several PN junctions, each made of materials with distinct band gaps, thereby optimizing the efficiency of the system.

The standard spectrum at the Earth’s surface is designated by the reference AM1.5G. This designation characterizes the dissipation of energy emitted by the Sun under the effect of atmospheric interaction, in comparison with the AM_0_ spectrum, which represents space, where no energy loss is observed. In this research, the light source used was the Sun, and the solar cells were illuminated assuming an AM1.5G solar spectrum, which is commonly used in terrestrial applications.

**Figure 5:**
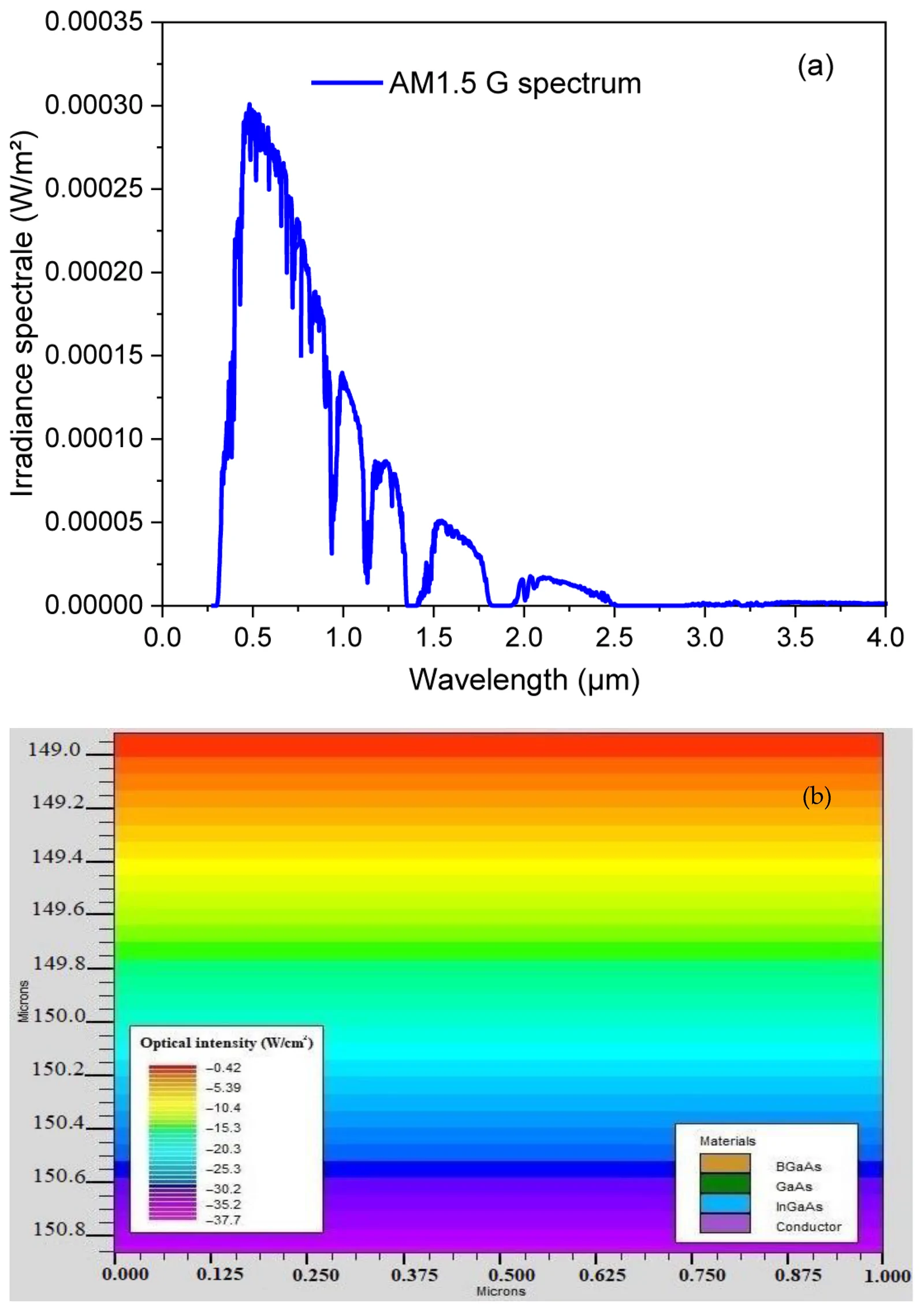
(**a**) AM1.5 spectrum used to illuminate the model; (**b**) optical intensity of different layers.

The AM1.5G spectrum is shown in Figure 5a, which is generated by ATLAS for our illumination. To optimize the use of the solar spectrum, it is essential that each sub-cell has a band gap greater than the one below it. In line with theoretical estimates, the system has a specific optical intensity depending on the various layers, as shown in Figure 5b, when exposed to this form of radiation. The upper B_x_Ga_1−x_As/GaAs cell is a state-of-the-art device that captures high-energy photons.

**Figure 6:**
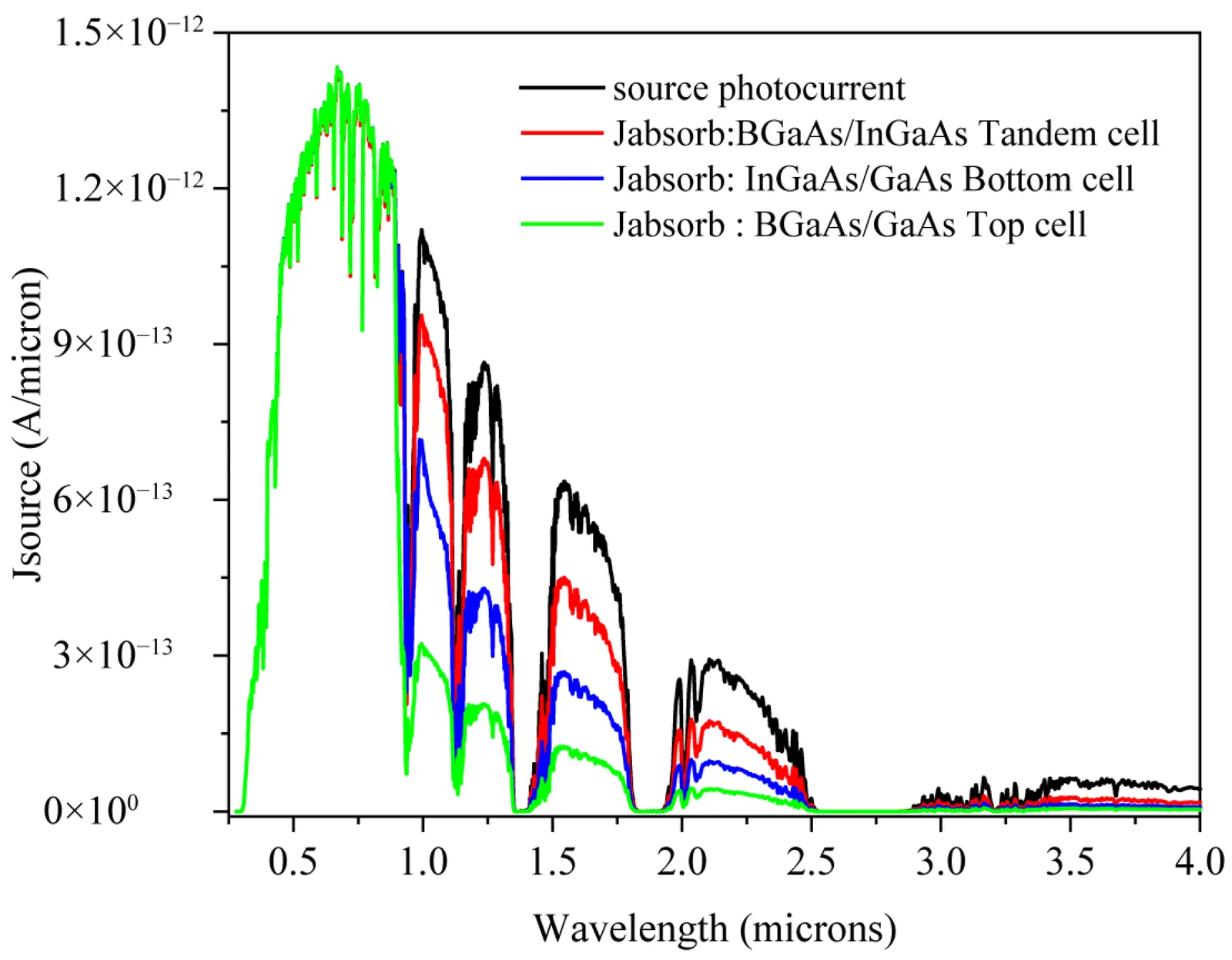
Processing of the spectrum of the current density absorbed by each sub-cell and by the tandem cell.

In optical spectroscopy, analysis of photogenerated currents (J_ph_) shows that the highest peak is generated in the upper layer, as shown in Figure 6. In contrast, the lower In_x_Ga_1−x_As/GaAs cell is used for photons with band gap energies close to infrared and visible light. It has been shown that BGaAs, GaAs, and InGaAs are remarkably efficient at absorbing photons with energies close to their band gaps. Site selection is based on regions where the presence of solar spectra (AM1.5G) is marked by high photon intensity. This selection aims to optimize the use of sunlight for energy production [38].

As shown in Table 1, the manufacture of two-terminal cells is technically more complex, as it requires the sub-cells to be aligned with each other. The two cells in a tandem configuration can be considered analogous to two diodes connected in series. It can therefore be deduced that the short-circuit current of the entire tandem cell is limited by the lowest short-circuit current (J_sc_) value of the sub-cell.

Furthermore, the open-circuit voltage of the tandem cell is the sum of the individual V_oc_ values of the sub-cells. The two-terminal solar cell is composed of two transparent conductive layers, which have been shown to reduce parasitic absorption and improve efficiency [39].

**Figure 7:**
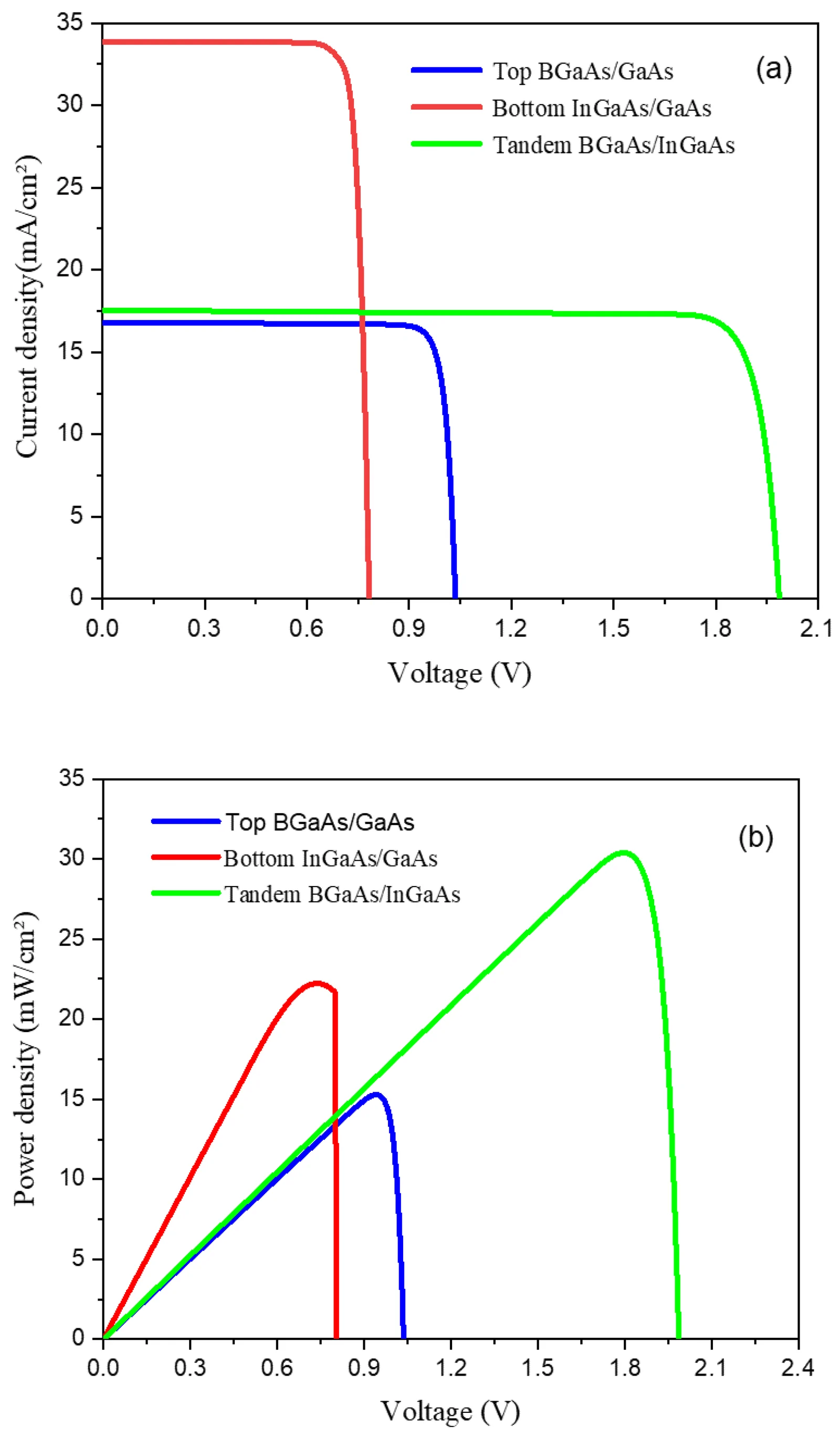
(**a**) Current density characteristics as a function of voltage of the three photovoltaic cells, measured under AM1.5 illumination, and (**b**) power characteristics as a function of voltage of the three photovoltaic cells, measured under AM1.5 illumination.

As shown in Figure 7, the current density and power characteristics as a function of voltage for a BGaAs/InGaAs tandem solar cell with a GaAs/GaAs tunnel diode are shown. For comparison, the isolated curves for the upper BGaAs/GaAs and lower InGaAs/GaAs cells are also shown. In this comparison, it appears that the InGaAs/GaAs SJSC has the highest J_sc_ but the lowest open-circuit voltage (V_oc_). In contrast, the BGaAs/GaAs SJSC has the lowest open-circuit current (I_sc_) but the highest open-circuit voltage (V_oc_), as shown in Table 2 and Figure 7. This shows that the optimized energy conversion efficiency is higher for the InGaAs/GaAs SJSC (22.57%) than for the BGaAs/GaAs SJSC (16.75%).

In this analysis, we focus on the performance of the DJSC BGaAs/InGaAs photovoltaic cell, which has an optimal efficiency of 30.40% [40], surpassing the efficiencies of the two sub-cells considered individually. In the course of this research, it was observed that J_sc_ was limited by the smallest of the sub-cells. The latter corresponds to the current adapted to that of the upper BGaAs/GaAs cell, as illustrated in Figure 7.

The DJSC has the highest open-circuit voltage. However, it is important to note that although DJSC achieves a higher open-circuit voltage than its respective individual sub-cells, it cannot achieve a conversion efficiency equal to the sum of the efficiencies of these sub-cells. This is because the current is limited to that of the lowest current due to the series connection. Figure 7a,b also illustrates the J_sc_ loss. In order to compensate for this loss, it is recommended that each junction be designed to generate an identical amount of photocurrent. In this configuration, even if photons in excess of the material’s band gap are present, only a minimum thickness is required to generate the required current [41]. Therefore, the experimental values of J_sc_, V_oc_, and FF, and the energy conversion efficiency of a DJSC of 17.50 mAcm^−2^, 1.99 V, 87.29%, and 30.40%, respectively, under AM1.5 G illumination, were compared with those reported in other related publications [42,43,44,45,46,47].

**Figure 8:**
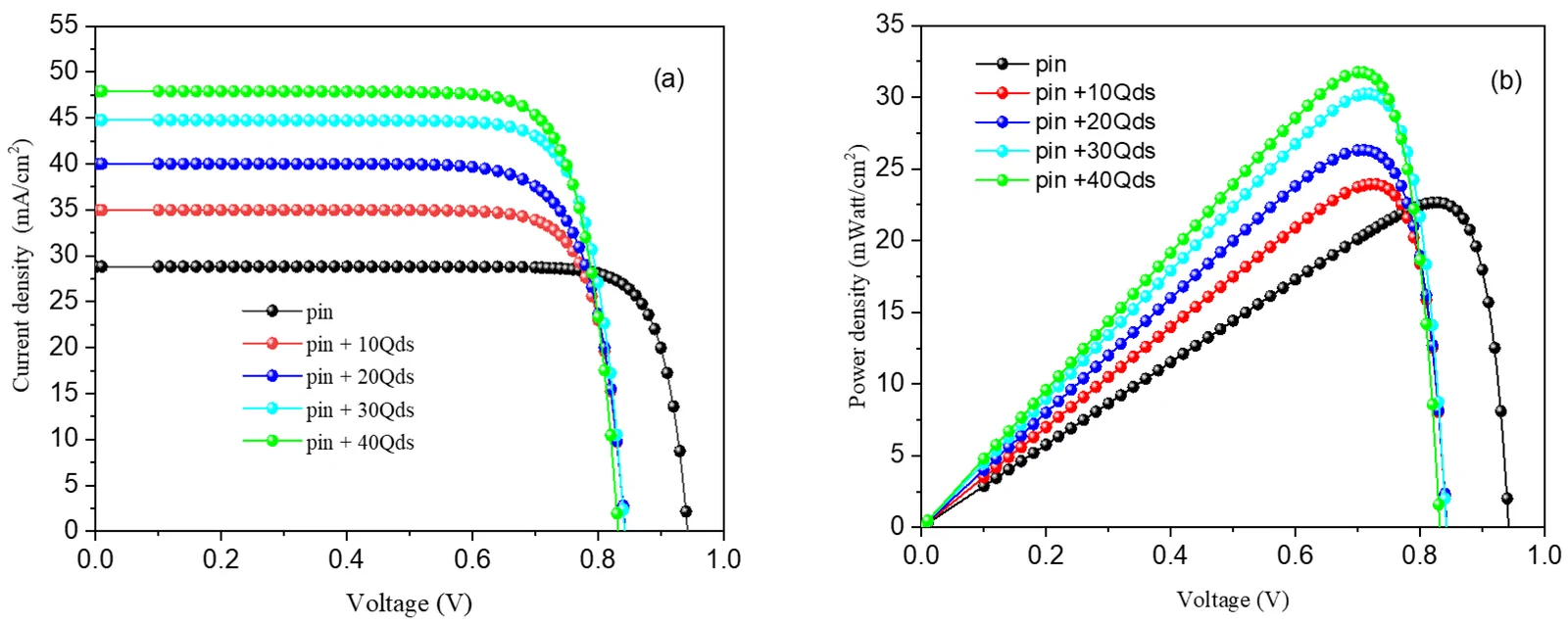
(**a**) J-V characteristics for different numbers of QWs and (**b**) power density versus voltage of a GaAs/B_x_In_y_Ga_1−x−y_As quantum well solar cell (QW layers = 0, 10, 20, and 40).

**Figure 9:**
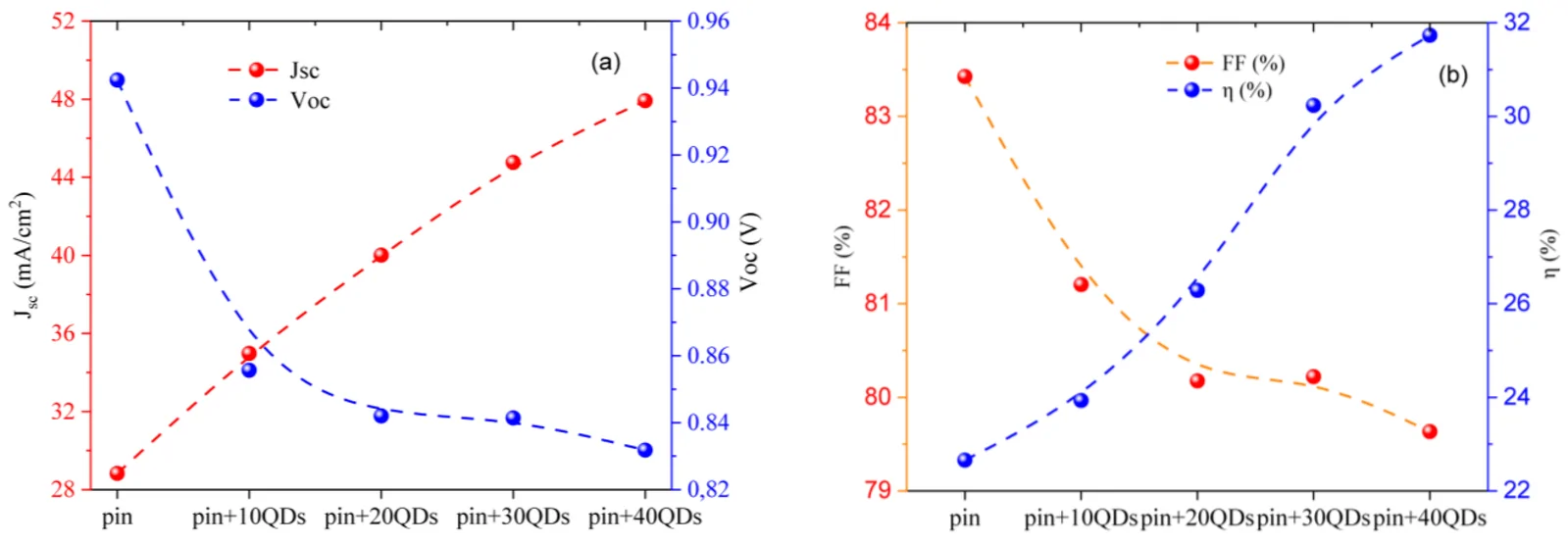
Effect of the number of quantum well layers on (**a**) J_sc_ and V_oc_, and (**b**) the factor and efficiency.

This study focuses on the analysis of a GaAs/B_x_In_y_Ga_1−x−y_As MQSC quantum well solar cell, whose X_bore_ and Y_indium_ alloy composition is similar to that of B_0.08_Ga_0.92_As/GaAs and GaAs/In_0.20_Ga_0.80_As solar cells, as well as the DJSC cell. In order to evaluate the performance of the well-optimized QWSC cell, a series of analyses is performed. These analyses focus on the thickness of each layer and the number of wells. In this regard, Figure 8a illustrates the current–voltage diagram J(V) of the GaAs/B_x_In_y_Ga_1−x−y_As quantum well solar cell as a function of the variable number of wells (0, 10, 20, 30, and 40), respectively. The analysis highlights an almost linear increase in short-circuit current J_sc_ as a function of the number of quantum well layers (see Figure 9). This increase is attributable to the absorption of additional electron–hole pairs by the quantum wells [48]. As demonstrated by several studies [49], including the aforementioned mathematical model, it has been observed that increasing the number of quantum wells leads to an increase in J_sc_ and a decrease in V_oc_, as illustrated in Figure 9.

Rigorous analyses have revealed that the increase in J_sc_ can be attributed to the presence of a low band gap material and the extension of the absorption edge across the quantum well region. This hypothesis, already stated by several authors, including [50], needs to be confirmed by further experiments. Furthermore, it has been observed that the increase in non-radiative dark current, resulting from the increase in the thickness of the depletion zone, leads to a reduction in V_oc_. This dynamic has the effect of undermining the competitive advantage of quantum wells. This reduction in V_oc_ in turn affects the FF, which also shows a downward trend. Studies have shown that increasing the number of wells reduces the overall performance of the solar cell. A similar trend has been observed in the context of III-V-based MQWSCs [51,52].

Furthermore, studies have shown that V_OC_ reduction is frequently correlated with an increase in barrier–well interfaces. In addition, empirical results have revealed a correlation between an increase in the number of quantum wells and an increase in J_sc_. This observation suggests an optimization of efficiency, as illustrated by the results presented in Figure 8 and Table 3, and we note that our simulation of this structure is compared with what has been found in the literature [53].

The evolution of the curve representing the power density as a function of voltage for an MQWSC quantum well solar cell (number of quantum wells equal to 0, 10, 20, or 40) is accurately illustrated in Figure 8b. The analysis revealed that the PIN solar cell, composed of 40 MQWSC layers, proved to be the most efficient structure, demonstrating the highest short-circuit current and power. This structure generates a power of approximately 31.73 mW/cm^2^.

Table 4 presents a performance comparison between the optimized QWSC cell and solar cells from other theoretical and experimental work available in the literature. The results of this simulation are consistent with recent experimental and theoretical studies. Our efficiency estimate of 31.73% is comparable to that presented in recent reports [54,55]. These results are particularly noteworthy given that previous research has shown lower performance [56,57]. This dynamic can be analyzed as follows: increasing the number of wells leads to a concomitant increase in photogeneration and carrier recombination.

On the one hand, the increase in the number of quantum wells leads to an improvement in the absorption volume. On the other hand, quantum wells (QWs) broaden the spectral range of photon absorption, which is lower in energy than the barrier material gap. As a result, we observe a concomitant increase in optical absorption and photogenerated carrier density. Furthermore, it should be mentioned that wells are confinement zones. In fact, they function as traps for carriers. This analysis leads to the conclusion that there is a positive correlation between the number of wells and the density of potential recombination sites.

As clearly shown in the results in Table 3 and Table 4, the increase in photogeneration leads to an increase in J_SC_ and an improvement in efficiency. This increase is due to the creation and extraction of more carriers. Furthermore, an increase in recombination causes a decrease in FF and V_OC_. The correlation between these two phenomena is responsible for the initial increase, followed by a decrease in J_SC_ and η. When photogeneration exceeds recombination, J_SC_ and η increase. However, when recombination dominates photogeneration, J_SC_ and η decrease (see Figure 9).

Analysis of the results obtained indicates that the performance of the DJSC B_x_Ga_1−x_As/In_y_Ga_1−y_As cell is comparable to that of the MQWSC GaAs/B_x_In_y_Ga_1−x−y_As cell for a common alloy composition value, namely x boron and y indium. The analysis shows that the DJSC cell has the highest V_oc_. This property is attributable to the contribution of two series junctions, which optimize the use of the solar spectrum and reduce thermalization losses. Each junction absorbs part of the spectrum, resulting in more efficient use of solar radiation.

The study conducted on the MQWSC cell revealed significant improvements in photocurrent (J_sc_), as well as a significant increase in efficiency and optimization of absorption at lower energies, thanks to the contribution of the wells. Furthermore, this solution is easier to implement than a true DJSC multi-junction. Thus, to optimize the performance of InGaAs/GaAs quantum wells, it is essential to incorporate a precise amount of boron, thereby effectively counterbalancing the compressive stresses caused by the addition of indium. This strategic approach aims to maximize yields, as it promotes increased carrier mobility within the BInGaAs alloy. The low defect density in the BInGaAs material could be one of the causes of this phenomenon.

The modeling strategy and design principles outlined in this study apply to all the structures investigated and provide a framework for optimizing advanced photovoltaic architectures. Our double-junction tandem has demonstrated its effectiveness in band gap engineering and current matching optimization. These promising results are directly applicable to triple- and quadruple-junction designs. They are particularly relevant to InGaP/GaAs/InGaAs configurations with matched crystal lattices or metamorphic structures incorporating dilute nitrides for lower sub-cells.

Our strategy focused on B_x_In_y_Ga_1−x−y_As quantum wells in GaAs, but the optimization principles can be applied to other material systems, such as GaAsP barriers with InGaAs wells for stress-balanced designs or dilute nitride wells (GaInNAs) for extended infrared absorption. Although the number, thickness, and composition of the wells must be optimized as system-specific, the underlying methodology is universally applicable.

This systematic approach, which encompasses the optimization of material composition, including thickness adjustment for current matching, provides a roadmap for the design of next-generation photovoltaic devices tailored to specific applications and performance requirements.

## 4. Conclusions

In this article, we present and compare several advanced solar cell architectures, including B_x_Ga_1−x_As/In_y_Ga_1−y_As tandem cells and PIN cells incorporating multiple GaAs/B_x_In_y_Ga_1−x−y_As quantum wells. This study reveals that each structure offers specific advantages that improve photovoltaic conversion efficiency. B_x_Ga_1−x_As/In_y_Ga_1−y_As tandem cells are characterized by their ability to exploit the entire solar spectrum by combining two B_x_Ga_1−x_As and In_y_Ga_1−y_As sub-cells with complementary band gaps. This approach enables them to achieve efficiencies of 30.40%, significantly surpassing those achieved by single-junction cells. Furthermore, PIN cells with GaAs/B_x_In_y_Ga_1−x−y_As quantum wells represent a more accessible technological alternative. The introduction of quantum structures in the intrinsic region broadens the absorption range and achieves high efficiencies of around 31.73%. These findings highlight the potential of these solutions for optimizing the performance of standard cells. The choice of cell architecture seems to be closely associated with the specific application, manufacturing limitations, and efficiency needs. The ingenious integration of new structures, such as quantum wells or multiple junctions, represents a promising avenue for expanding the capabilities of conventional photovoltaic technologies.

Although this work presents a complete theoretical optimization using validated simulation tools, experimental demonstration of the proposed structures is an essential step to confirm their practical viability. The 40-layer GaAs/B_x_In_y_Ga_1−x−y_As quantum well structure requires a controlled thickness at the nanoscale, which is achievable with modern growth systems, as well as rigorous optimization of growth conditions to minimize interface roughness and point defects. Our simulated yields (31.73% for quantum well cells, 30.40% for tandems) are consistent with experimental reference values reported for similar III-V devices, confirming the accuracy of the modeling. A complete experimental validation includes: optimization of epitaxial growth, structural characterization, device fabrication, performance characterization, and model refinement. This experimental program not only validates our specific designs, but it also establishes a reliable modeling framework for the future development of III-V photovoltaic devices. The expected strong agreement between simulation and experiment—based on the physical models validated in ATLAS and the consistency of our results with published data—reinforces the practical utility of this theoretical study as a guide for experimental work and will accelerate the development of high-efficiency quantum well and tandem photovoltaic devices for terrestrial and space applications.

## Figures and Tables

**Table 2:** Comparison of the output parameters of each cell under AM1.5G illumination.

Solar Cell	J_sc_ (mA/cm^2^)	V_oc_ (V)	FF (%)	η (%)
BGaAs/GaAs SJ	16.82	1.03	81.59	16.75
InGaAs/GaAs SJ	33.82	0.81	86.17	22.57
BGaAs/InGaAs Tandem cell (DJSC)	17.50	1.99	87.29	30.40

**Table 3:** Important parameters of a standard solar cell and a GaAs/B_x_In_y_Ga_1−x−y_As quantum well solar cell with different numbers of QW layers.

Number of QWs	Jsc (mA/cm^2^)	Voc (V)	FF (%)	η (%)
pin no QWs [53]	27.52	1.03	88.53	25.2
pin no QWs	28.83	0.94	83.43	22.66
pin + 10QWs	34.98	0.88	81.20	23.93
pin + 20QWs	40.01	0.85	80.17	26.28
pin + 30QWs	44.76	0.84	80.22	30.24
pin + 40QWs	47.92	0.83	79.63	31.73

**Table 4:** A performance comparison to the reported work will now be conducted.

Reference	Cell Materials	MQWs	η (%)
[56]	InSb	GaAS	29.30
[57]	GaAs	AlGaAs	28.72
[58]	GaAs	InAs	33.58
[59]	GaSb	GaAs	30.00
This work	GaAs	BInGaAs	31.73

## Data Availability

The original contributions presented in the study are included in the article. Further inquiries can be directed to the corresponding authors.

## Nomenclature Abbreviations

GaAs	Gallium Arsenide
BGaAs	Boron Gallium Arsenide
InGaAs	Indium Gallium Arsenide
AlGaAs	Aluminum Gallium Arsenide
BInGaAs	Boron Indium Gallium Arsenide
TJ	Tunnel Junction
DJSC	Dual Junction Solar Cell
MJSC	Dual Junction Solar Cell
SJ	Single Junction
MQWSC	Multi-Quantum Well Solar Cell
J_sc_	Short Current Density
V_oc_	Open-Circuit Voltage
FF	Fill Factor
η	Efficiency
E_c_, E_v_	Energy Levels of the Conduction and Valence Bands
E_g_	Band Gap Energy
hν	Photon Energy
τ_n_	Electron Lifetime
τ_p_	Hole Lifetime
ε_r_	Relative Permittivity
χ	Affinity
N_c_	Electron Density of State
N_v_	Hole Density of State
μ_n_	Electron Mobility
μ_p_	Hole Mobility
AM	Air Mass
AM1.5	Air Mass 1.5 or Standard Test Conditions

## References

[1] Humaidan R.M., Dahham A.T., Majeed Z.N. (2022). Designed and Simulation of AlGaAs:GaAs Thin Film Solar Cell Using PC1D Program. NeuroQuantology.

[2] Al-Ezzi A.S., Anas S.M., Ansari M.N.M. (2025). Characterization and Computational Modeling of Flexible Freestanding GaAs-Based Solar Cells. Eng. Rep..

[3] Wang Y., Zhang X., Guo M. (2015). Needle Profile Grating Structure for Absorption Enhancement in GaAs Thin Film Solar Cells. Opt. Laser Technol..

[4] Machín A., Márquez F. (2024). Advancements in photovoltaic cell materials: Silicon, organic, and perovskite solar cells. Materials.

[5] Rathore N., Panwar N.L., Yettou F., Gama A. (2021). A Comprehensive Review of Different Types of Solar Photovoltaic Cells and Their Applications. Int. J. Ambient Energy.

[6] Efaz E.T., Rhaman M.M., Imam S.A., Bashar K.L., Kabir F., Mourtaza M.E., Sakib S.N., Mozahid F.A. (2021). A Review of Primary Technologies of Thin-Film Solar Cells. Eng. Res. Express.

[7] Torchynska T.V. (2002). III–V Material Solar Cells for Space Application. Semicond. Phys. Quantum Electron. Optoelectron..

[8] Angadi R.V., Revanasiddesh B., Vineet Kumar P.K. (2019). A Review on Different Types of Materials Employed in Solar Photovoltaic Panel. Int. J. Eng. Res. Technol..

[9] Deshpande R.A. (2021). Advances in Solar Cell Technology: An Overview. J. Sci. Res..

[10] Verma M., Mishra G.P. (2023). Analytical Model of InP QWs for Efficiency Improvement in GaInP/Si Dual Junction Solar Cell. Phys. Status Solidi A.

[11] Verma M., Sahoo G.S., Mishra G.P. (2025). Bandgap Engineering and Passivation in Top and Bottom Layer of GaAsP/Si Tandem Solar Cell for Efficiency Improvement. J. Opt..

[12] Warepam D., Singh K.J., Dhar R.S. (2024). CNT-Based Enhanced GaAs/InAs Multiple Quantum Well Solar Cell. J. Comput. Electron..

[13] France R.M., Geisz J.F., Song T., Olavarria W., Young M., Kibbler A., Steiner M.A. (2022). Triple-Junction Solar Cells with 39.5% Terrestrial and 34.2% Space Efficiency Enabled by Thick Quantum Well Superlattices. Joule.

[14] Suchikova Y., Nazarovets S., Konuhova M., Popov A.I. (2025). Binary Oxide Ceramics (TiO_2_, ZnO, Al_2_O_3_, SiO_2_, CeO_2_, Fe_2_O_3_, and WO_3_) for Solar Cell Applications: A Comparative and Bibliometric Analysis. Ceramics.

[15] Al-Ezzi A.S., Ansari M.N.M. (2022). Photovoltaic Solar Cells: A Review. Appl. Syst. Innov..

[16] Green M.A., Emery K., Hishikawa Y., Warta W., Dunlop E.D. (2013). Solar Cell Efficiency Tables (Version 41). Prog. Photovolt. Res. Appl..

[17] Ramirez Torres J.D., Briones Viera R.E., Obispo A.E. (2025). Numerical Simulation of Performance in GaAs/InGaAs Quantum Well Intermediate-Band Bifacial Solar Cells Using Spectral Albedo. Int. J. Eng..

[18] Gottschalch V., Leibiger G., Benndorf G. (2003). MOVPE Growth of BxGa1−xAs, BxGa1−x−yInyAs, and BxAl1−xAs Alloys on (001) GaAs. J. Cryst. Growth.

[19] Barnes J., Tsui E.S.M., Barnham K.W.J., McFarlane S.C., Button C., Roberts J.S. (1997). Steady-State Photocurrent and Photoluminescence from Single Quantum Wells as a Function of Temperature and Bias. J. Appl. Phys..

[20] Barnham K.W.J., Ballard I., Barnes J., Connolly J.P., Griffin P., Kluftinger B., Nelson J., Tsui E.S.M., Zachariou A. (2002). Quantum Well Solar Cells. Phys. E.

[21] Wu J., Walukiewicz W., Shan W., Yu K.M., Ager J.W., Haller E.E., Lu H., Schaff W.J. (2002). Effects of the Narrow Band Gap on the Properties of InN. Phys. Rev. B.

[22] Ekins-Daukes N.J., Adams J., Ballard I.M., Connolly J.P., Barnham K.W.J., Mazzer M., Nelson J., Rohr C., Yoshida M., Witzigmann B., Osinski M., Arakawa Y. (2009). Physics of Quantum Well Solar Cells. Physics and Simulation of Optoelectronic Devices XVII.

[23] Jani O., Ferguson I., Honsberg C., Kurtz S. (2007). Design and Characterization of GaN/InGaN Solar Cells. Appl. Phys. Lett..

[24] Thilagam A., Singh J., Stulik P. (1998). Optimizing Gallium Arsenide Multiple Quantum Wells as High-Performance Photovoltaic Devices. Sol. Energy Mater. Sol. Cells.

[25] Shan W., Walukiewicz W., Ager J.W., Haller E.E., Geisz J.F., Friedman D.J., Olson J.M., Kurtz S.R. (1999). Band Anticrossing in GaInNAs Alloys. Phys. Rev. Lett..

[26] Pelá R.R., Marques M., Teles L.K. (2015). Comparing LDA-1/2, HSE03, HSE06 and G_0_W_0_ Approaches for Band Gap Calculations of Alloys. J. Phys. Condens. Matter.

[27] Kaschner A., Lüttgert T., Born H., Hoffmann A., Egorov A.Y., Riechert H. (2001). Recombination Mechanisms in GaInNAs/GaAs Multiple Quantum Wells. Appl. Phys. Lett..

[28] Adachi S. (2009). Properties of Semiconductor Alloys: Group-IV, III–V and II–VI Semiconductors.

[29] Wang X.D., Hu W.D., Chen X.S., Lu W. (2008). Dark Current Simulation of InP/In_0.53_Ga_0.47_As/InP p-i-n Photodiode. Opt. Quantum Electron..

[30] Saidi F., Hassen F., Maaref H., Bouzaïene L. (2004). Comparative Optical Study of GaAs_1−X_N_X_/GaAs and B_X_Ga_1−X_As/GaAs Epilayers. IEE Proc. Optoelectron..

[31] Hidouri T., Saidi F., Al-Shahri B.M. (2021). Tuning Spontaneous Emission in BInGaAs/GaAs QWs by Varying the Growth Temperature: Above 1.2 µm Emission and Solar Cells Application. Opt. Quantum Electron..

[32] Arbia M.B., Smiri B., Demir I., Moumanis K., Maaref H., Mezrag F., Ajjel R. (2022). Theoretical Analyses of the Carrier Localization Effect on the Photoluminescence of In-Rich InGaAs Layer Grown on InP. Mater. Sci. Semicond. Process..

[33] Hidouri T., Saidi F., Maaref H., Bouzaïene L. (2016). LSE Investigation of the Thermal Effect on Band Gap Energy and Thermodynamic Parameters of BInGaAs/GaAs Single Quantum Well. Opt. Mater..

[34] Benbahouche L., Hadji Idjdarene S. (2022). An Improved Performance and High Efficiency DJ III–V Tandem Solar Cell with the Impact of Quantum Tunneling. Appl. Sol. Energy.

[35] Hamila R., Saidi F., Rodriguez P.H., Auvray L., Monteil Y., Maaref H. (2010). Growth temperature effects on boron incorporation and optical properties of BGaAs/GaAs grown by MOCVD. J. Alloys Compd..

[36] Tuley R.S., Nicholas R.J. (2010). Band gap dependent thermophotovoltaic device performance using the InGaAs and InGaAsP material system. J. Appl. Phys..

[37] Shockley W., Queisser H.J. (1961). Detailed Balance Limit of Efficiency of p-n Junction Solar Cells. J. Appl. Phys..

[38] Singh K.J., Sarkar S.K. (2012). Highly Efficient ARC Less InGaP/GaAs DJ Solar Cell Numerical Modeling Using Optimized InAlGaP BSF Layers. Opt. Quantum Electron..

[39] Jacobsson T.J., Pazoki M., Hagfeldt A., Edvinsson T. (2015). Goldschmidt’s Rules and Strontium Replacement in Lead Halogen Perovskite Solar Cells: Theory and Preliminary Experiments on CH_3_NH_3_SrI_3_. J. Phys. Chem. C.

[40] Bouzid F., Pezzimenti F., Dehimi L., Della Corte F.G., Hadjab M., Hadj Larbi A. (2019). Analytical Modeling of Dual-Junction Tandem Solar Cells Based on an InGaP/GaAs Heterojunction Stacked on a Ge Substrate. J. Electron. Mater..

[41] Sabri S., Malek R., Kassmi K. (2023). Improvement Efficiency of Solar Cells Using III–V Dual Junction: InGaP/GaAs. Key Eng. Mater..

[42] Jomen R., Tanaka F., Akiba T., Ikeda M., Kiryu K., Matsushita M., Maenaka H., Dai P., Lu S., Uchida S. (2018). Conversion Efficiencies of Single-Junction III–V Solar Cells Based on InGaP, GaAs, InGaAsP, and InGaAs for Laser Wireless Power Transmission. Jpn. J. Appl. Phys..

[43] Geisz J.F., Kurtz S., Wanlass M.W., Ward J.S., Duda A., Friedman D.J., Olson J.M., McMahon W.E., Moriarty T., Kiehl J. (2007). High-Efficiency GaInP/GaAs/InGaAs Triple-Junction Solar Cells Grown Inverted with a Metamorphic Bottom Junction. Appl. Phys. Lett..

[44] Kim Y., Park S., Nguyen T.T., Jeon J., Chun B.S., Lee S.J. (2025). High-Efficiency Flexible GaAs/InGaAs Dual-Junction Solar Cells Fabricated by Metallic Nanoparticle-Based Wafer Bonding. Small Struct..

[45] Sahoo G.S., Mishra G.P. (2016). Design and Modeling of an Efficient Metamorphic Dual-Junction InGaP/GaAs Solar Cell. Opt. Quantum Electron..

[46] Zhao X.F., Aierken A., Heini M., Tan M., Wu Y.Y., Lu S.L., Hao R.T., Mo J.H., Shen X.B., Xu Y. (2020). Degradation Characteristics of Electron- and Proton-Irradiated InGaAsP/InGaAs Dual Junction Solar Cell. Sol. Energy Mater. Sol. Cells.

[47] Zidani I., Bensaad Z., Hafaifa L., Hamza A., Hafaifa A. (2025). Modeling and Efficiency Enhancement of InGaP/GaAs Dual-Heterojunction Solar Cells Using AlGaAs and AlGaInP Emitters. Semicond. Sci. Technol..

[48] Aissat A., Benyettou F., Nacer S., Vilcot J.P. (2017). Modeling and Simulation of Solar Cells Quantum Well Based on SiGe/Si. Int. J. Hydrogen Energy.

[49] Prabhu S., Pandey S.K., Chakrabarti S. (2022). Innovative Structural Engineering of Sustainable and Environment-Friendly Cu_2_ZnSnS_4_ Solar Cell for over 20% Conversion Efficiency. Int. J. Energy Res..

[50] Rimada Herrera J.C., Hernández L., Connolly J.P., Barnham K.W.J. (2007). Conversion Efficiency Enhancement of AlGaAs Quantum Well Solar Cells. Microelectron. J..

[51] Bushnell D.B., Tibbits T.N.D., Barnham K.W.J., Connolly J.P., Mazzer M., Ekins-Daukes N.J., Roberts J.S., Hill G., Airey R. (2005). Effect of Well Number on the Performance of Quantum-Well Solar Cells. J. Appl. Phys..

[52] Lynch M.C., Ballard I.M., Bessière A., Durose K., Peaker A.R., Barnham K.W.J., Ekins-Daukes N.J., Roberts J.S., Hill G., Airey R. (2005). Spectral response and IV characteristics of large well number multi quantum well solar cells. J. Mater. Sci..

[53] Slimi D., Hamila R., Saidi F., Helali A., Madani M., Dhaou M.H., Ilkay D. (2025). Simulation and Improvement Performance of PN (BGaAs/GaAs) and PIN (GaAs/BGaAs/GaAs) Junction Solar Cells. J. Ovonic Res..

[54] Soley S.S., Verma S., Khatri N., Pokhriyal S. (2024). Advancing Efficiency: Comprehensive Strategies for Minimizing Optical and Electrical Losses in Group III–V Compound Tandem Solar Cells for Future Photovoltaic Technology. Eng. Res. Express.

[55] Aissat A., Bestam R., Boubakeur M., Vilcot J.-P. (2022). Development of the Multi-Quantum Wells Structures Based on InxGa1−xAs1−yNy/GaAs for Solar Cells Applications. Micro Nanostruct..

[56] Aissat A., Benyettou F., Vilcot J.P. (2017). Electrical and Optical Properties of InSb/GaAs QDSC for Photovoltaic. Int. J. Hydrogen Energy.

[57] Laznek S., Meftah A., Meftah A., Sengouga N. (2018). Semi-Analytical Simulation and Optimization of AlGaAs/GaAs PIN Quantum Well Solar Cell. Appl. Sol. Energy.

[58] Islam M.J., Chowdhury M.I.B. (2024). Investigation of an InGaN Based Quantum Well Solar Cell Using Silvaco TCAD. J. Adv. Elect. Eng. Dev..

[59] Islam M.J., Mostafa S., Chowdhury M.I.B. (2020). SILVACO TCAD Implementation of Dual Junction Quantum Well Solar Cell. J. Altern. Renew. Energy Sources.

